# Persistent elevation of plasma vitamin B12 is strongly associated with solid cancer

**DOI:** 10.1038/s41598-021-92945-y

**Published:** 2021-06-25

**Authors:** Valentin Lacombe, Floris Chabrun, Carole Lacout, Alaa Ghali, Olivier Capitain, Anne Patsouris, Christian Lavigne, Geoffrey Urbanski

**Affiliations:** 1 Department of Internal Medicine and Clinical Immunology, Angers University Hospital, Angers, France; 2 Department of Biochemistry and Genetics, Angers University Hospital, Angers, France; 3 Ouest Institute of Cancerology, Angers, France; 4 Department of Internal Medicine and Clinical Immunology, Angers University Hospital, 4 rue Larrey, 49000 Angers, France

**Keywords:** Biomarkers, Cancer, Metabolic disorders

## Abstract

Elevated plasma vitamin B12 has been associated with solid cancers, based on a single B12 measurement. We evaluated the incidence of solid cancers following B12 measurement in patients with persistent elevated B12, compared to patients without elevated B12 and to patients with non-persistent elevated B12**.** The study population included patients with at least two plasma B12 measurements without already known elevated-B12-related causes. Patients with elevated plasma B12 (≥ 1000 ng/L) at first measurement (n = 344) were matched for age and sex with patients having 2 normal B12 measurements (< 1000 ng/L) (NN group, n = 344). The patients with elevated plasma B12 at first measurement were split into 2 groups, according to the presence (EE group, n = 144) or the absence (EN group, n = 200) of persistent elevated plasma B12 at second measurement. We compared the cancer-free survival during 60 months between the groups after adjustment for the other elevated-B12-related causes in a survival competing risk model. Compared to the NN group, a persistent elevated plasma B12 ≥ 1000 ng/mL was strongly associated with the occurrence of solid cancer (HR 5.90 [95% CI 2.79–12.45], p < 0.001), contrary to non-persistent plasma B12 elevation (p = 0.29). These results could help to select patients in whom the screening for solid cancers would be of interest.

## Introduction

The plasma vitamin B12 (B12) measurement is mainly conducted to detect vitamin B12 deficiency, but the incidental finding of elevated B12 is not uncommon^1,2^. An elevated B12 is generally defined as a level higher than the upper limit of the normal range, around 1000 ± 100 ng/L (738 ± 73.8 pmol/L)^2–4^. Elevated B12 has been associated with various diseases^1,5,6^: liver diseases^7–10^, myeloid blood malignancies^10–13^, chronic renal failure^3,14^, autoimmune or inflammatory diseases^3^, Gaucher disease^15^. An association between elevated B12 and solid cancers has been demonstrated by two population-based cohort studies^16,17^ and persists after adjustment for the other elevated-B12-related causes^1^.

No consensus exists concerning a diagnostic strategy in case of incidental finding of elevated B12^5,18,19^. Some causes can be explored with simple investigations (blood count, renal function evaluation, liver cytolysis and cholestasis parameters, and liver echography) but solid cancer exploration often requires imaging or endoscopic examinations. The risk of solid cancer seems insufficient to systematically perform these invasive and expansive investigations in case of incidental discovery of elevated B12. Patients in whom active screening should be discussed need to be better targeted to allow early diagnosis and limit unnecessary investigations.

In studies evaluating the association between solid cancer and elevated B12, a single measurement was sufficient to define an elevated B12 level^1,16,17^. In our own daily practice, we observed spontaneous normalizations of elevated B12 level after the resolution of acute disorders (severe infections, acute inflammatory state due to immune or inflammatory diseases). We therefore hypothesized that acute conditions could have temporarily raised the B12 level. On the contrary, elevated B12 levels encountered in some cancers could be correlated with the tumor mass or the granulocytic immune response^5,6,20^. Consequently, if elevated B12 levels are caused by some cancers, elevated B12 should persist as long as the cancer persists.

The objective of this study was to evaluate the proportion of incident solid cancers in patients with persistent elevated B12 level, compared to patients without elevated B12 and to patients with non-persistent elevated B12.

## Patients and methods

### Ethics and statement for study checklist

This study was approved by the bioethical committee of Angers University Hospital (n°2019/105) and has been conducted in accordance with the Declaration of Helsinki. Patients gave informed consent. We applied the strengthening the reporting of observational studies in epidemiology (STROBE) statement to observational studies.

### Study population

This study included patients aged 18 years and over, admitted to Angers University Hospital between January 2007 and May 2015. Patients were required to have undergone two B12 measurements at 2 different times (T1 and T2) at 1 to 48 months intervals. Assays were initially performed to search for B12 deficiencies. Assays realized in intensive care and maternity units were excluded because of the metabolic changes observed in these patients^21–23^.

Patients with identified elevated-B12-related causes including a solid cancer before/at T1 were excluded. The elevated-B12-related causes were: acute liver disease (acute elevation of transaminases to more than 2 times normal) or chronic liver disease (dysmorphic ultrasound appearance, persistent biological signs of hepatocellular insufficiency, and/or histology suggestive of cirrhosis), severe chronic renal failure (MDRD clearance ≤ 30 mL/min/1.73 m^2^), autoimmune or inflammatory disease, Gaucher disease, myeloid blood malignancy, active solid cancer, and cancer treated over the last 5 years^1,2,6^. Patients with pernicious anemia or those supplemented with vitamin B12 were also excluded. In the case of solid cancer diagnosed between T1 and T2, patients were excluded if the T2 assay was performed at more than 1 month after cancer treatment initiation.

### Plasma vitamin B12 assay

The B12 measurements were centralized in the biochemistry laboratory of Angers University Hospital. The tests were carried out on an immunoanalytical system ADVIA Centaur (SIEMENS HEALTHCARE DIAGNOSTICS Inc. Tarrytown, NY, USA) with ADVIA Centaur VB12 reagents.

An elevated B12 level was defined as ≥ 1000 ng/L^1,4,24–26^.

In patients with three or more B12 measurements and at least one B12 ≥ 1000 ng/L, T1 was selected as the first test with B12 ≥ 1000 ng/L. In the absence of B12 ≥ 1000 ng/L, T1 was randomly selected between the first and the penultimate B12 measurement. T2 was the test immediately following T1 with at least 1 month between T1 and T2. As the study aimed at comparing persistent and non-persistent elevated B12, patients who had only the last measurement ≥ 1000 ng/L were excluded.

### Groups’ constitution

Patients with B12 ≥ 1000 ng/L at T1 were selected and matched with a control group. Control patients were randomly selected among those with B12 < 1000 ng/L at T1 and T2, with a ratio 1:1, matching for sex, age and the number of B12 measurements (2, 3, ≥ 4) during the study period. These control patients defined the NN (normal/normal) group. Then, the patients with B12 ≥ 1000 ng/L at T1 were split into 2 groups, those having B12 ≥ 1000 ng/L at T2 (EE group) and those having B12 < 1000 ng/L at T2 (EN group). Thus, 3 groups of patients were defined according to B12 status: EE (elevated/elevated), EN (elevated/normal) and NN. As explained above, patients in the EE or EN groups could have had previous normal B12 measurements before the first elevated one (T1).

### Collected data

All patient records have been fully reviewed.

The following general data were collected: sex, age, B12 levels, dates of B12 measurement, and death date. We collected the incident elevated-B12-related causes, including solid cancers, which appeared within 60 months following T1, and their date of occurrence.

Solid cancers were defined as non-hematological malignancies. The site of primary cancer (according to the International Classification of Diseases for Oncology classification), presence of metastasis and site of metastasis were collected.

### Statistical methods

The quantitative data were presented in medians and quartiles and compared using a Student t test or an ANOVA. The categorical data were presented as absolute values and as percentages and were compared using Chi-squared test.

Time-to-event curves for incident solid cancer were presented as Kaplan–Meier curves and were compared with a log-rank test. Follow-up was limited at 60 months. Loss of follow-up was censored. The influence of covariate (including age, sex, and all the elevated-B12-related causes) on the occurrence of solid cancer was evaluated with a survival competing risk model (package cmprsk from R) with the death as the competing risk. The proportional hazard assumption was checked with 2 different methods: graphically by plotting the log(minuslog) curves and by studying the interaction with time. The alpha risk was 5%. The hazard ratios (HR) were presented with a confidence interval of 95%. The analyses were carried out using GRAPHPAD Prism v6.01 (GRAPHPAD SOFTWARE, La Jolla, CA, USA) and R software (version 3.5.1, R-project.org, Vienna, Austria).

## Results

### Description of the population

**Figure 1:**
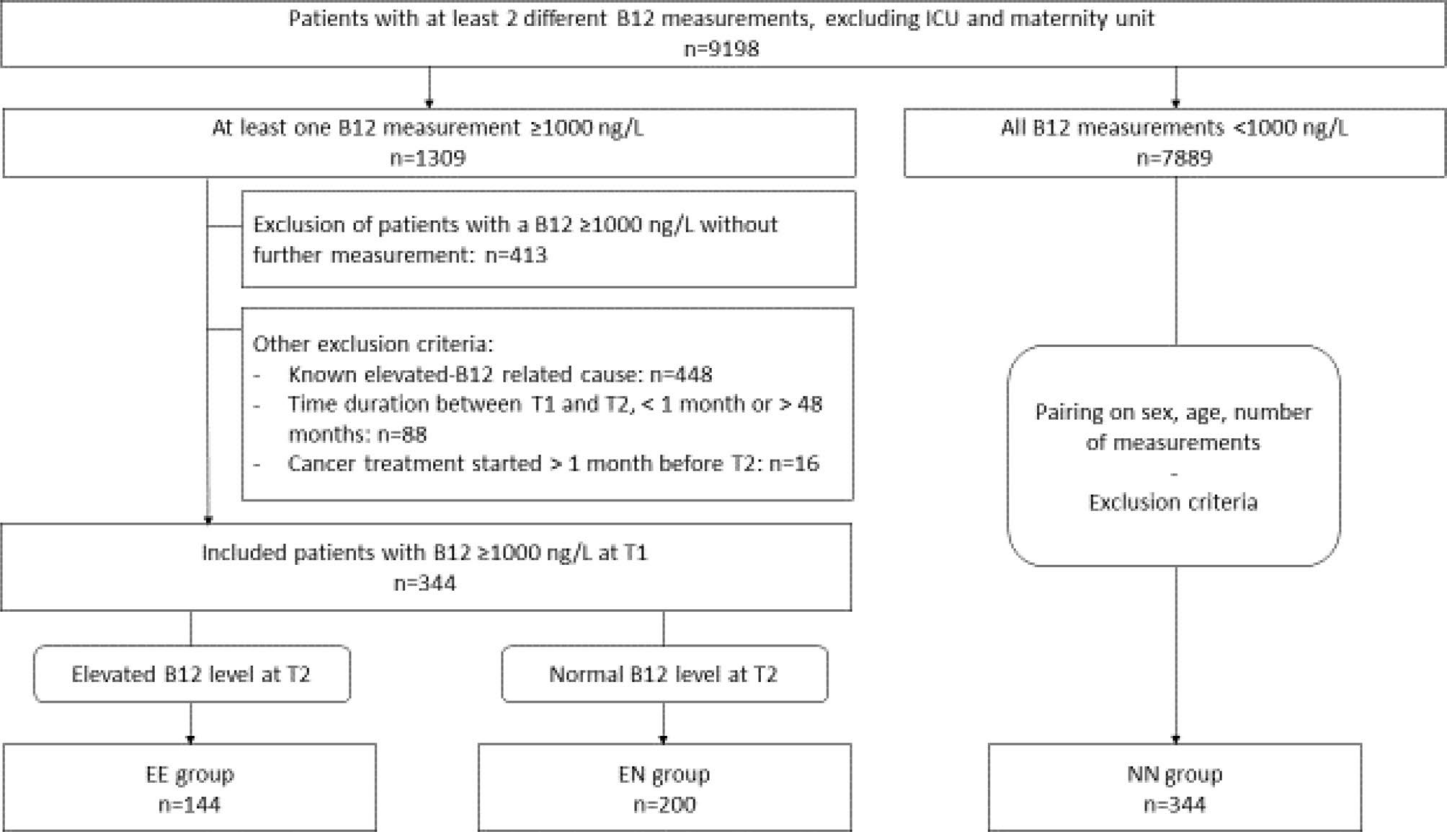
Flowchart.

Between January 2007 and May 2015, 9,198 patients underwent at least 2 measurements of B12 in our center, excluding the intensive care and maternity units. Among these patients, plasma B12 ≥ 1000 ng/L at T1 were found in 344 patients without any known elevated-B12-related causes. Among these 344, 144 (41.9%) patients had a B12 ≥ 1000 ng/L at T2 (EE group) and 200 (58.1%) patients had a B12 < 1000 ng/L at T2 (EN group). The 344 patients in the NN group were randomly selected from the 7,889 patients with plasma B12 < 1000 ng/L at T1 and T2. The flowchart is detailed in Fig. 1.

The study population included 688 patients with a median age of 79 [64–86] years. There were 256/688 men (37.2%), and the median time from T1 to the last hospitalization or consultation was 3.2 [1.5–5.2] years.

The age of the patients was similar in the 3 groups (Table 1). The proportion of men was higher in the EE group, but with no significant difference (p = 0.06). B12 at T1 was higher in the EE group than in the EN group (p < 0.001). The death rate was higher in the EE group than in the other groups (p < 0.001).

**Table 1:** Comparison of the 3 groups.

	EE group (n = 144)	EN group (n = 200)	NN group (n = 344)	p-value
**Age (at T1)**	78 [68–87]	80 [60–86]	79 [64–86]	0.74
**Sex (men)**	64 (44.4%)	64 (32.0%)	128 (37.2%)	0.06
**Number of realized measurements**				
2 measurements	86 (59.8%)	109 (54.5%)	195 (56.7%)	0.85
3 measurements	29 (20.1%)	49 (24.5%)	74 (21.5%)	
≥ 4 measurements	29 (20.1%)	42 (21.0%)	75 (21.8%)	
**Interval T1-T2 (days)**	252 [81–504]	272 [92–523]	250 [119–496]	0.82
**Plasma vitamin B12 at T1 (ng/L)**	1521 [1206–2000]	1177 [1063–1414]	364 [287–509]	< 0.001
**Plasma vitamin B12 at T2 (ng/L)**	1544 [1226–2000]	613 [427–804]	370 [281–495]	< 0.001
**Time between T1 and last admission (years)**	2.7 [0.7–4.7]	3.1 [1.5–5.3]	3.6 [1.8–5.3]	0.01
**Death**	47 (32.6%)	32 (16.0%)	44 (12.8%)	< 0.001
**Diseases diagnosed during the follow-up period**				
At least 1 elevated-B12-related cause	75 (52.1%)	34 (17.0%)	38 (11.0%)	< 0.001
Chronic liver disease	32 (22.2%)	11 (5.5%)	5 (1.5%)	< 0.001
Severe renal failure^a^	0 (0%)	2 (1.0%)	0 (0%)	0.09
Autoimmune/inflammatory disease	3 (2.1%)	1 (0.5%)	4 (1.2%)	0.43
Myeloid blood malignancy	25 (17.4%)	6 (3.0%)	13 (3.8%)	< 0.001
Solid cancer	30 (20.8%)	12 (6.0%)	14 (4.1%)	< 0.001
Without metastasis	14 (9.7%)	7 (3.5%)	9 (2.6%)	0.002
With metastasis	16 (11.1%)	5 (2.5%)	5 (1.5%)	< 0.001
Lymphoid blood malignancy	2 (1.4%)	2 (1.0%)	3 (0.9%)	0.87

The 3 groups were compared by ANOVA or Chi-squared test, as appropriate.

^a^Creatinine clearance with MDRD formula ≤ 30 mL/min/1.73 m^2^.

### Incident causes of elevated B12

At least one cause of elevated B12 was diagnosed in the follow-up of 75/144 (52.1%) patients in the EE group, 34/200 (17.0%) in the EN group, and 38/448 (11.0%) in the NN group (p < 0.001, Table 1).

Among the causes of elevated B12, incident myeloid blood malignancies and chronic liver diseases were more frequent in the EE group (p < 0.001 and p < 0.001, respectively). The frequency of myeloid blood malignancy did not differ between the EN and NN groups, and chronic liver disease was more common in the EN group than in the NN group.

Incident solid cancer was diagnosed in 30/144 (20.8%) patients in the EE group, 12/200 (6.0%) in the EN group and 13/344 (3.8%) in the NN group (p < 0.001). Compared with the reference NN group, the EE group was associated with the occurrence of solid cancers with or without metastases (p < 0.001 and p < 0.001, respectively), contrary to the EN group (p = 0.56 and p = 0.38, respectively). Solid cancers and metastases sites are presented in Table S1, supplemental material.

### Solid cancer-free survival adjusted for age, sex, and other elevated-B12-related causes

**Figure 2:**
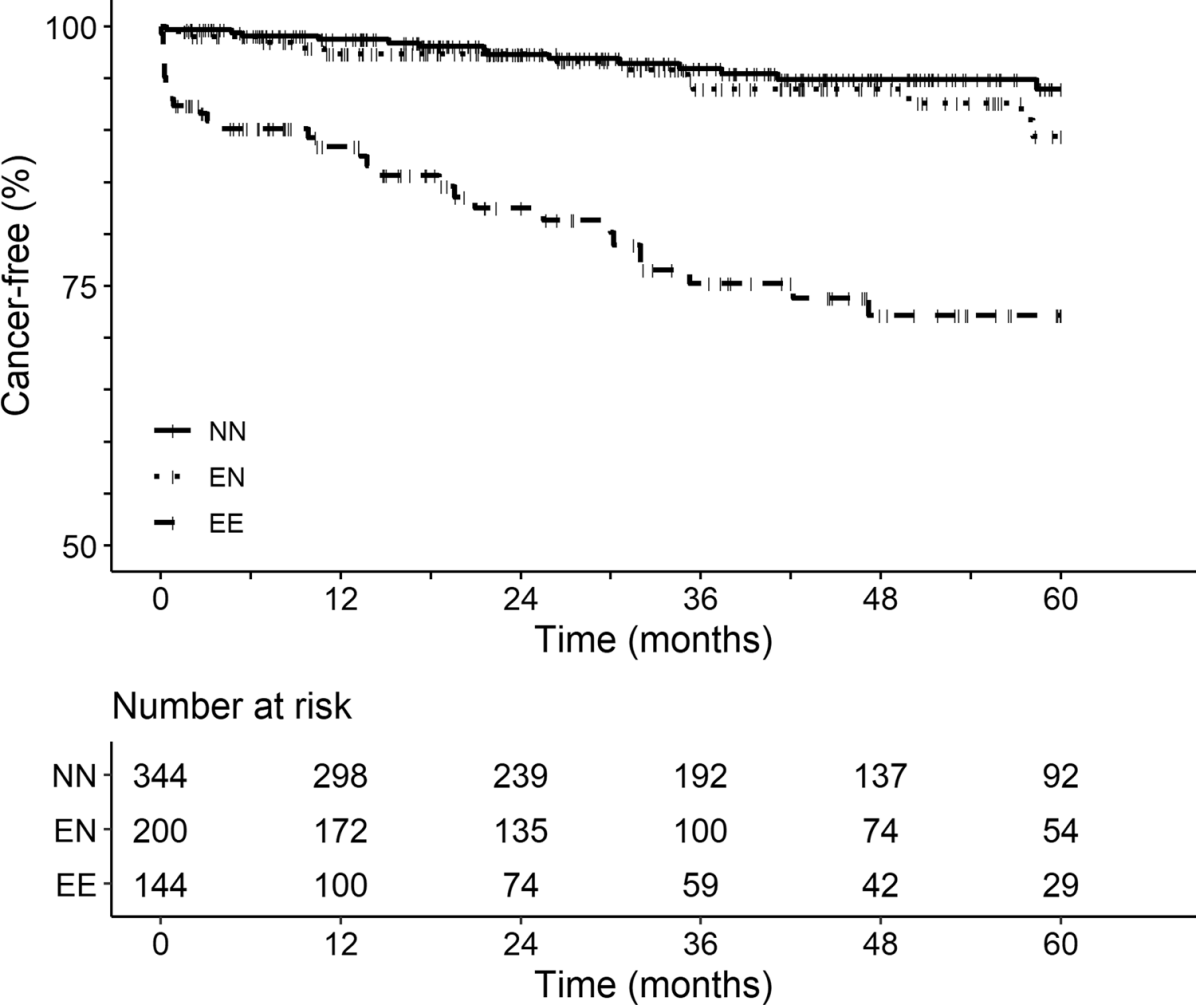
Cancer-free survival in the 3 groups. Events only referred to solid cancers and not to blood malignancies. Loss of follow-up was censored. The influence of covariate (including age, sex, and all the elevated-B12-related causes) on the occurrence of solid cancer was evaluated with a survival competing risk model (package cmprsk from R) with the death as the competing risk.

The cancer-free survival significantly differed between the 3 groups (p < 0.001, Fig. 2). The difference was linked to a more frequent occurrence of incident solid cancer in the EE group (p < 0.001 compared with EN and NN groups), as the EN and NN groups did not differ (p = 0.28).

Compared with the NN group as a reference, only the EE group was significantly associated with incident cancer with HR 5.90 [95% CI 2.79–12.45] (p < 0.001), contrary to the EN group (HR 1.52 [95% CI 0.70–3.30], p = 0.29) (Table 2).

**Table 2:** Strength of association between incident solid cancers within 60 months and the 3 groups in an adjusted competing risk survival model with the death as a competing risk.

	Hazard ratio (95%CI)	p-value
**Group**		
NN	Reference	–
EN	1.52 (0.70–3.30)	0.29
EE	5.90 (2.79–12.45)	< 0.001
Age^a^	1.01 (0.23–1.93)	0.98
Sex (men)	1.27 (0.71–2.26)	0.42
Myeloid blood malignancies	0.28 (0.07–1.16)	0.08
Lymphoid blood malignancies	2.69 (0.53–13.53)	0.23
Chronic liver diseases	1.39 (0.57–3.40)	0.46
Severe chronic kidney failure	0.41 (0.05–3.53)	0.41
Autoimmune/inflammatory diseases	1.14 (0.17–7.56)	0.89

^a^Age as dichotomous variable with the median age as a threshold (< or ≥ 79 years).

## Discussion

In the case of incidental finding of elevated B12, the relevance of an active search for solid cancer necessitating invasive and expensive examinations remains debated^5,18,19^. Indeed, few studies analyzed the incidence of solid cancers following the incidental finding of elevated B12 and no studies linked elevated B12 to solid cancer after adjusting for other causes of elevated B12. Our study aimed at evaluating the incidence of solid cancers in patients with persistent elevated B12, in comparison with patients without elevated B12 and those with non-persistent elevated B12.

The association between elevated B12 and solid cancers was demonstrated by two population-based studies: a B12 > 800 pmol/L (1084 ng/L) was associated with a diagnosis of cancer in the following year with a Standardized Incidence Ratio of 6.3 [95% CI 5.7–6.9] in a Danish cohort^16^, and a B12 between 800 and 1000 pmol/L (1084–1355 ng/L) was associated with an Incidence Rate Ratio of 2.9 [95% CI 2.4–3.5] in a British cohort^17^. However, to our knowledge, no study has evaluated this association according to the persistence of this B12 elevation. In our study, an increased risk of cancer was associated with patients having a persistent elevated B12 (group EE) but not with those showing transient elevated B12 (group EN). For the first time, we demonstrate that the risk of incident solid cancer in the case of transiently elevated B12 is similar to that of patients without elevated B12, whereas this risk is strongly increased in the case of persistent elevated B12.

The incidence of solid cancers in the EE group argues for checking the persistence of B12 elevation to justify investigations in case of incidental finding of B12 elevation. The occurrence of solid cancers in 20.8% of patients in the EE group could justify careful clinical surveillance and raises the question of carrying out complementary investigations. In our study, a significant number of solid cancers were diagnosed more than one year after T1 (51.5% in the EE group). This raises the question of the potential interest in repeating investigations 1 year later.

This is interesting for also exploring the other causes related to elevated B12. Indeed, the frequency of incident diagnoses of chronic liver diseases (32/144, 22.2%) and myeloid blood malignancies (25/144, 17.4%) in the EE group also confirmed the interest in screening these pathologies in case of persistent elevated B12^5,18,19^.

Some authors suggested that the development of solid cancers could be secondary to the elevated B12^27,28^. Indeed, vitamin B12 is the cofactor of methionine synthase, which is implicated in methylation reaction and in the synthesis of purine bases^29,30^, and these functions are crucial in tumor-initiating cells and cell proliferation^30,31^. On the contrary, we think that certain cancers would be, directly or indirectly, responsible for B12 elevation. Moreover, Arendt et al. showed that the SIR of cancers was higher within the year following B12 measurement than in subsequent years^16^. This supports the presence of undiagnosed subclinical cancer rather than a hypothetical role of the elevated B12 in the development of cancer.

In this study, we limited the analyses to cancers occurring within 60 months following B12 measurement, because it appeared to be difficult to establish the causal link between a subclinical solid cancer and an elevated plasma B12 beyond a period of 5 years^32–35^.

The mechanism of elevated B12 in case of solid cancer is poorly understood^5,6,20^. The prognostic nature of elevated B12 in solid cancers suggested the question of a possible link with the tumor mass or the capacity for proliferation^20^. The first hypothesis consists in the secretion of a tumor mediator increasing the bioavailability of vitamin B12, promoting the synthesis of nucleic acids by cancer cells. The second hypothesis is that of the release of haptocorrins by the granulocytic cells involved in the anti-tumor response.

Our study has some limitations. The incidence of elevated-B12-related causes could be underestimated due to the retrospective and non-interventional nature of the study. However, this would identically impact the 3 groups. We cannot exclude that the elevated B12 level may have prompted some physicians to look for underlying cancer in patients of the EE and EN groups, but this limitation is imbalanced by the prolonged follow-up (3.2 [1.5–5.2] years in the whole population, with a longer follow-up in the NN group), which could have enabled detection of cancers that have not been actively searched initially. We are not able to exclude loss of information due to the fact that follow-up was carried out in several centers. Nevertheless, the bulk of the study data came from the university hospital, which is the main healthcare facility in the region, so the risk of information loss appears to be reduced and is not biased between the groups. We set the period of at least 1 month between the 2 measurements to allow normalization of B12 in case of acute transient elevation. However, the study methodology did not allow for determination of the most suitable time to perform this control. The higher proportion of men in the EE group could have biased the risk of incident cancer, since men are more exposed to environmental risk factors^36^. To avoid this, we included the sex of patients as a variable of adjustment in the survival model and, finally, sex did not significantly influence the occurrence of cancer at 60 months. We also observed a higher mortality in the EE group, which could possibly bias the incidence of solid cancers. We anticipated this bias using a survival competing risk model with the death as the competing risk. Patients in our study were hospitalized, so the results need to be confirmed in an outpatient population. Our results are support looking for the cause of an incidental finding of elevated B12, however we cannot propose B12 measurement as a screening marker for solid cancer at this stage.

## Conclusion

The persistence of elevated B12 was associated with a high incidence of solid cancer at 60 months, in contrast to a transient B12 elevation. Solid cancers represent one of the main diagnoses found in patients with unexplained and persistent elevated B12. An unexplained elevated B12 level should be confirmed later by a second measurement, which could help to identify patients in whom the screening for solid cancers would be of interest.

## Supplementary Information

Supplementary Table S1.
