# Supplementary material for: Persistent elevation of plasma vitamin B12 is strongly associated with solid cancer
Source: Sci Rep. 2021 Jun 25;11:13361. doi: 10.1038/s41598-021-92945-y (PMC8233305; doi:10.1038/s41598-021-92945-y)
Supplement: Supplementary file 1 — Supplementary Table S1. [file 41598_2021_92945_MOESM1_ESM.docx]

**Table S1.** Sites of solid cancer and metastases

|  | **EE group** | **EN group** | **NN group** |
| --- | --- | --- | --- |
| **Number of patients** | **144** | **200** | **344** |
| **Solid cancers** | **30 (20.8%)** | **12 (6.0%)** | **14 (4.1%)** |
| **Sites of primary cancers *(ICD-O classification)*** |  |  |  |
| Colon/rectum *(C18-20)* | 3 (10.0%) | 0 (0%) | 6 (42.8%) |
| Liver *(C22)* | 5 (16.7%) | 2 (16.7%) | 0 (0%) |
| Pancreas *(C25)* | 4 (13.3%) | 0 (0%) | 0 (0%) |
| Skin *(C44, melanoma)* | 0 (0%) | 2 (16.7%) | 2 (14.3%) |
| Skin *(C44, epidermoid carcinoma)* | 0 (0%) | 3 (25.0%) | 0 (0%) |
| Lungs *(C34)* | 4 (13.3%) | 2 (16.7%) | 0 (0%) |
| Prostate *(C61)* | 1 (3.3%) | 0 (0%) | 1 (7.1%) |
| Kidney *(C64-65)* | 0 (0%) | 0 (0%) | 0 (0%) |
| Breast *(C50)* | 1 (3.3%) | 1 (8.3%) | 2 (14.3%) |
| Urinary bladder/Urothelium *(C66-67)* | 2 (6.7%) | 1 (8.3%) | 0 (0%) |
| Esophagus and stomach *(C15-16)* | 2 (6.7%) | 1 (8.3%) | 1 (7.1%) |
| ENT *(C00-14 and C30-32)* | 1 (3.3%) | 0 (0%) | 2 (14.3%) |
| Unknown primary | 4 (13.3%) | 0 (0%) | 0 (0%) |
| Others | 2 (6.7%) | 0 (0%) | 0 (0%) |
| **Solid cancers with metastases** | **16 (11.1%)** | **5 (2.5%)** | **5 (1.5%)** |
| **Sites of metastases** |  |  |  |
| Brain | 2 (6.7%) | 1 (8.3%) | 0 (0%) |
| Liver | 5 (16.7%) | 3 (25.0%) | 0 (0%) |
| Bones | 5 (16.7%) | 0 (0%) | 1 (7.1%) |
| Lungs | 2 (6.7%) | 2 (16.7%) | 0 (0%) |
| Peritoneum | 3 (10.0%) | 0 (0%) | 2 (14.3%) |
| Lymph nodes | 3 (10.0%) | 3 (25.0%) | 2 (14.3%) |
| Others | 1 (3.3%) | 0 (0%) | 0 (0%) |

Notes: ICD-O: Internal Classification of Diseases for Oncology
